# Comparison of Microstructure and Properties of In-Situ TiN- and WC-Reinforced NiCrBSi Composite Coatings Prepared by Plasma Spraying

**DOI:** 10.3390/ma11112182

**Published:** 2018-11-04

**Authors:** Linlin Zhu, Jing Wang, Xianqiang Deng, Yanchun Dong, Yong Yang, Dongyang Li

**Affiliations:** 1 Tianjin Key Laboratory of Materials Laminating Fabrication and Interface Control Technology, School of Materials Science and Engineering, Hebei University of Technology, Tianjin 300130, China; zhulinlin608@126.com (L.Z.); wj_hebut@126.com (J.W.); deng228732@foxmail.com (X.D.); yangyonghebut@163.com (Y.Y.); 2 Department of Chemistry and Materials Engineering, University of Alberta, Edmonton, AB T6G 2L5, Canada; dongyang@ualberta.ca

**Keywords:** TiN, NiCrBSi, WC, plasma spraying, wear resistance

## Abstract

In this study, NiCrBSi-30 wt.% TiN composite (NTC) coating was produced on carbon steel via plasma spraying, with NiCrBSi-30 wt.% WC composite (NWC) coating as the comparison object. The microstructure and phase constituents of the composite coatings were characterized using scanning electron microscopy (SEM) coupled with energy dispersive spectroscopy (EDS) techniques, transmission electron microscopy (TEM) and x-ray diffraction (XRD). Atomic force microscopy (AFM) was used to measure electronic work functions. The microhardness and wear performance of coatings were also investigated. The average microhardness of the NTC and NWC coatings was 1000 HV and 850 HV, respectively. In addition, the NTC coating had a wear volume loss of 0.8118 mm^3^, less than 1.4772 mm^3^, the volume loss of the NWC coating. This was due to the presence of TiN in the form of nanograins in the composite coating and tighter binding to the matrix.

## 1. Introduction 

Plasma spraying technology is widely used in industrial production to prepare various protective coatings [1,2]. These coatings, attached to mechanical component surfaces, have high hardness and good abrasion and corrosion resistance, which can prevent the components from being severely worn and corroded and increase the machinery lifespan [3]. Typically, the materials used for plasma spraying are metal-based composites, which consist of a metal matrix with good toughness and reinforcing ceramic particles [4]. By integrating the toughness of the metal and the hardness of the ceramic particles, the wear resistance of composites is significantly improved. The most representative metal matrix composites are usually Ni-based or Co-based alloys containing SiC, TiC, WC, La_2_O_3_, or Cr_3_C_2_ [5,6,7,8]. Relatively speaking, Ni-based alloy powders have been widely studied and applied due to their excellent self-fluxing performance, wear resistance, and low price [9]. In addition, WC has high hardness and good wettability compared with other carbides (SiC, TiC) [10,11,12]. Therefore, most of the current research focuses on WC-strengthened Ni-based alloy coatings. Zhang et al.’s [13] research results showed that compared with NiCrBSi coating, the microhardness of NWC coating was significantly enhanced, and rolling contact fatigue performance was improved. Serresde’s [14] study also proved that the addition of WC enhanced the wear resistance of NiCrBSi coating. These studies show that adding WC to Ni-based alloys can improve the microstructure and hardness, as well as the wear resistance and rolling contact fatigue properties. However, the failure of the NWC coating is related to the intergranular cracks generated by the separation of WC/W_2_C and Ni-based matrix crystals. These cracks are due to the thermal stress caused by the rapid cooling and mismatch of the thermal expansion coefficient of the WC phase with the matrix phase [15]. In addition, some WC phases decarburize to form lower-hardness W_2_C due to overheating by thermal spraying jets, and further decarburization forms a softer W phase, resulting in lower hardness of the coating [16]. Therefore, this will restrict its potential application in the engineering field.

Except for the above-mentioned reinforcing phase particles, TiN has been widely used for the enhancement of coatings for its high hardness, wear resistance, and good corrosion resistance [17,18,19]. Previous studies prepared NiCrBSi-TiN composite coating by mechanically mixing Ni-based powders with TiN powders as raw coating materials, in which TiN was decomposed due to the high temperature. In addition, it is difficult to deposit TiN coating using TiN powders because of its high melting point (2950 K) [20]. In this experiment, the NTC coating was formed by spraying Ti and NiCrBSi powders, in which TiN-reinforced particles were formed by in situ reaction of titanium powder and nitrogen [19,20,21]. The TiN particles in the composite coating are in a fully molten state and bond well with the matrix to avoid pyrolysis during the spraying process [17,18]. This work characterized the microstructures and mechanical properties of the NTC coating, and compared it with the NWC coating.

## 2. Experimental Procedure

### 2.1. Sample Preparation

**Figure 1:**
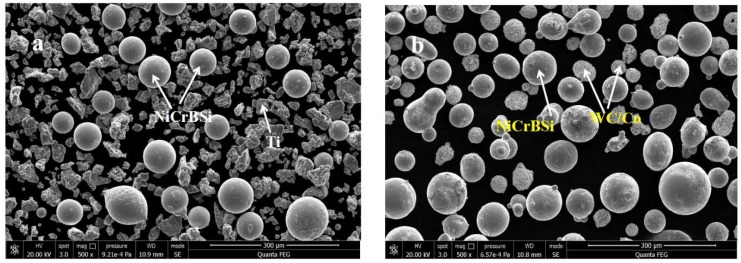
Morphology of mixing powders: (**a**) NiCrBSi–Ti; (**b**) NiCrBSi–WC/Co.

In this investigation, NiCrBSi and Ti powders as feed-stock materials for preparing NiCrBSi–TiN composite (NTC) coating were commercially available from Beijing Xing Rong Yuan Technology Corporation of China. The two kinds of powders were mixed in a ball mill for half an hour at a weight ratio of 7:3. NiCrBSi-WC/Co powder was available on the market. Figure 1 shows the morphology of the two kinds of composite powder. The size of NiCrBSi powder with spherical shape was about 45–100 μm. Its chemical composition is presented in Table 1. The Ti powder had irregular shapes, and the WC particles had a spherical shape but were rough, with particle sizes in the range of 25–45 μm, as shown in Figure 1b. AISI1045 steel (0.42–0.50% C, 0.17–0.37% Si, 0.50–0.80% Mn, ≤0.25% Cr, and the balance Fe) with a size of 10 × 10 × 12 mm^3^ was used as substrate, and the surface of the substrate was roughened by alumina sandblasting before spraying.

A GP-80 type plasma spraying system (Yeyuan Spraying Corp., Taixing, China) was used to deposit coatings. Argon (purity: 99.9999%) was used as primary plasma gas, while nitrogen (purity: 99.99%) was used as powder carrier gas. The thicknesses of the two composite coatings were controlled between 250 and 270 μm. A NiCrBSi bonding bottom layer was also prepared with a thickness of 50–80 μm.

### 2.2. Coating Characterization

Scanning electron microscopy (SEM; Hitachi S-4800/TMP, Hitach Ltd., Tokyo, Japan) was used to observe the microstructure and wear morphology, while energy dispersive spectroscopy (EDS; EDAX-AMETEK, Hitach Ltd., Tokyo, Japan) was applied to analyze the composition and distribution of the elements. The phase composition of the coating was identified by a D8 x-ray diffractometer (Bruker Scientific Technology Ltd., Karlsruhe, Germany). At room temperature, the interface morphology and electron work function (EWF) of the composite coatings were measured using a scanning Kelvin probe (SKP 5050, KP Technology Ltd., Wick, UK). The section microhardness of the coating was measured using an HMV-2T hardness tester (Shimadzu Ltd., Tokyo, Japan). The test parameters were as follows: 200 g load and 15 s dwell time. 

### 2.3. Tribological Tests 

At room temperature, tribological tests were performed with an SFT-2M tribometer (Zhongke Kaihua Technology Development Corp., Lanzhou, China). To ensure the reliability of the results, five specimens were tested for every test result, and the wear volume loss was averaged. Prior to testing, each specimen was ground and polished. The chosen counterpart was an Si_3_N_4_ ball with a diameter of 4 mm. Tribological experiments were performed using a load of 30 N, 400 rad/min sliding speed, rotation radius 3 mm, and duration 30 min. Wear volume was evaluated by using a Nano Indenter XP type mechanical performance microprobe (MTS Systems Corp., Eden Prairie, MN, USA) connected to a computer to measure the wear trace profile. Wear morphology was observed using scanning electron microscopy.

## 3. Results and Discussion

### 3.1. Microstructure Analysis

**Figure 2:**
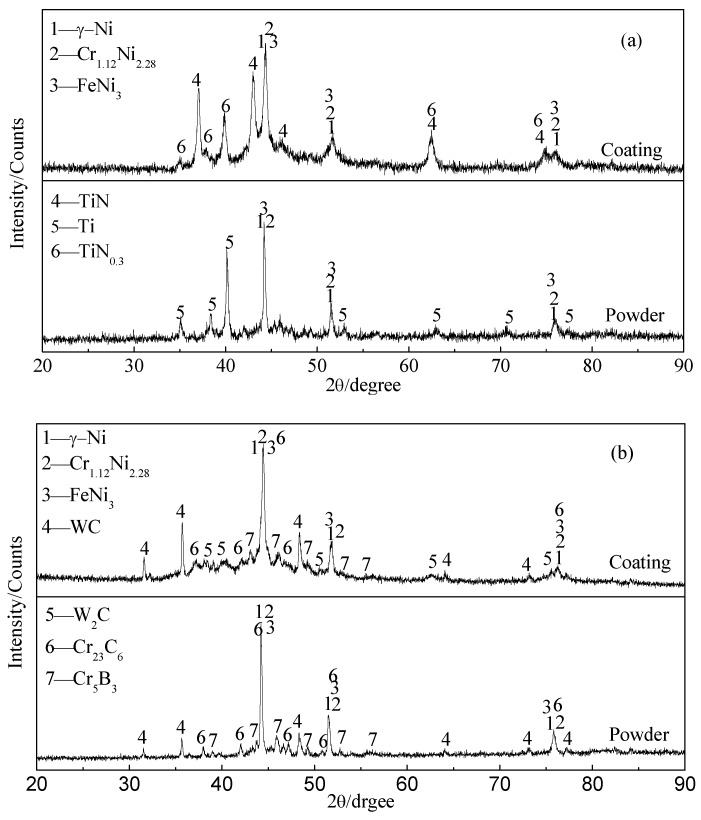
XRD patterns of original powders and composite coatings: (**a**) NiCrBSi–TiN composite (NTC); (**b**) NiCrBSi–WC composite (NWC).

The x-ray diffraction patterns of starting NiCrBSi–Ti mixed powder (Figure 1a) and NiCrBSi–TiN composite (NTC) coating are shown in Figure 2a. The NTC coating was composed of γ-Ni (PDF# 65-0380), FeNi_3_ (PDF# 38-0419), Cr_1.12_Ni_2.88_ (PDF# 65-5559), TiN (PDF# 38-1420), and TiN_0.3_ (PDF# 41-1352). Among these phases, TiN and TiN_0.3_ appearing in coatings should be attributed to the in situ reaction between Ti powders and N_2_ in air and plasma gas [19,20]. Figure 2b shows the x-ray diffraction patterns of plasma spraying NiCrBSi–WC composite (NWC) coating and NiCrBSi–WC/Co mixed powder. Compared with powders, new peaks of W_2_C (peak 5, PDF# 65-3896) were observed in the NWC coating and some peaks of WC (PDF# 65-4539) disappeared. The WC decomposition temperature was about 1250 °C [12]. During plasma spraying, WC was decomposed into softer W_2_C due to the high temperature [16,22].

**Figure 3:**
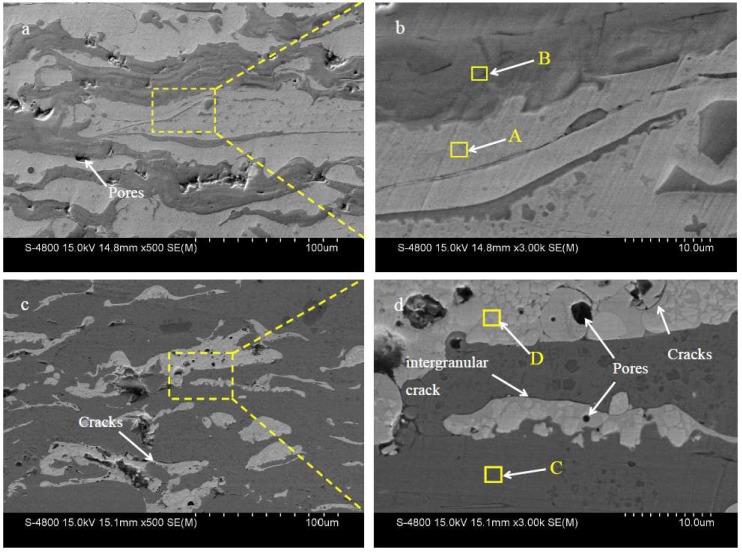
Cross-sectional morphologies of composite coating: (**a,b**) NTC coating; (**c,d**) NWC coating.

The cross-sectional structures of the NTC coating produced by plasma spraying are shown in Figure 3a,b. The NTC coating had the typical lamellar structure due to sprayed particles fully melting and flattening during plasma spraying [23]. The NTC coating displayed good bonding state, and no large cracks were observed. However, some small pores and irregular pits were observed. In addition, the deep-gray region (marked B) scattered throughout the light-gray region (marked A). Based on XRD (Figure 2a) and EDS (Table 2) analysis, the light-gray region was NiCrBSi alloy and the deep-gray region was composed of TiN and TiN_0.3_. The interface bonding state between the TiN and Ni-rich phase was very good and no cracks were observed. This is due to the fact that TiN is in a fully molten state during spraying and freezes together with NiCrBSi alloy powders, which is in molten state, too, promoting bonding with NiCrBSi alloy substrates [24]. This will also increase the strength of the coating while increasing the wear resistance.

Figure 3c,d shows the structure of NWC coating. Based on XRD (Figure 2b) and EDS (Table 2) analysis, the light-gray region (marked C in Figure 3d) shows the γ-Ni, FeNi_3_, and Cr_1.12_Ni_2.88_, while the white region (marked D in Figure 3d) shows the WC and W_2_C phases. The WC and W_2_C phases were evenly distributed in the NiCrBSi alloy matrix. There were lots of small pores and some cracks in the NWC coating. Transverse cracks were mainly distributed in the interface between the WC/W_2_C and Ni-rich phase; some cracks existed in the matrix areas. Figure 3d is an enlarged view of Figure 3c, in which the cracks between the WC/W_2_C phase and the Ni-rich phase can clearly be observed. The causes of crack formation are as follows: On the one hand, residual stress appeared due to rapid solidification and a thermal expansion mismatch between WC/W_2_C and Ni-rich phases. The thermal stress generated transverse interlaminar cracks [25]. On the other hand, WC has a high melting point and exhibits an unmelted or semimelted state in the coating, such that the bond between the WC/W_2_C and Ni-rich phase is reduced, resulting in crack formation. [26]. When the coating was stressed, WC was easily detached from the matrix, which influenced the mechanical properties of the coating.

**Figure 4:**
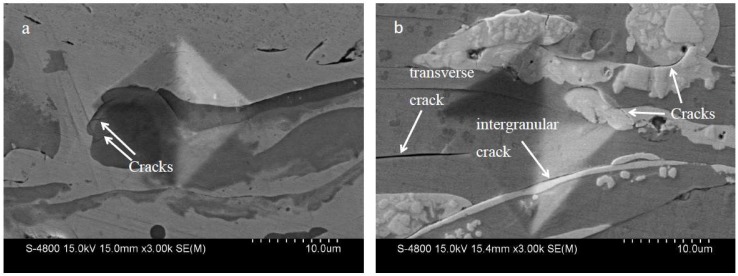
Cross-sectional SEM images of indentation morphology: (**a**) NTC coating; (**b**) NWC coating.

Figure 4 shows SEM images of indentation morphology on cross-sections of two kinds of composite coatings. The indentation profile was clear and no big cracks that extended around were present in either coating. However, two fine cracks in the TiN phase were observed in the NTC coating and the WC/W_2_C phase also broke. This is because the TiN and WC/W_2_C were hard phases with poor toughness. Compared with the complete bonding of the TiN phase and the Ni-rich phase in the NTC coating, there was a significant crack between the WC/W_2_C and Ni-rich phases in the NWC coating. It was easily found that the bonding state of the Ti-rich phase and the Ni-rich phase was stronger than that of the WC/W_2_C and Ni-rich phases. The dimensions of indentation in Figure 4b are bigger than those in Figure 4a, showing that NTC coating had higher hardness than NWC coating.

**Figure 5:**
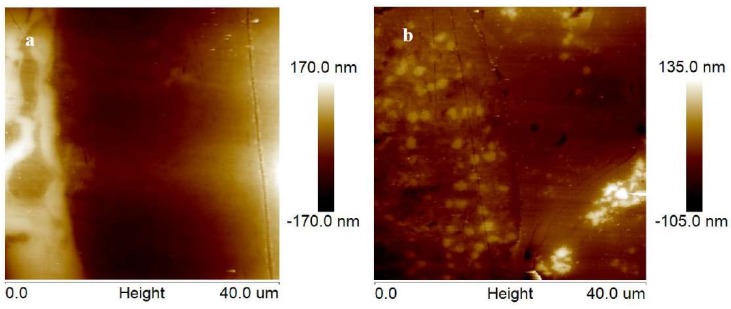

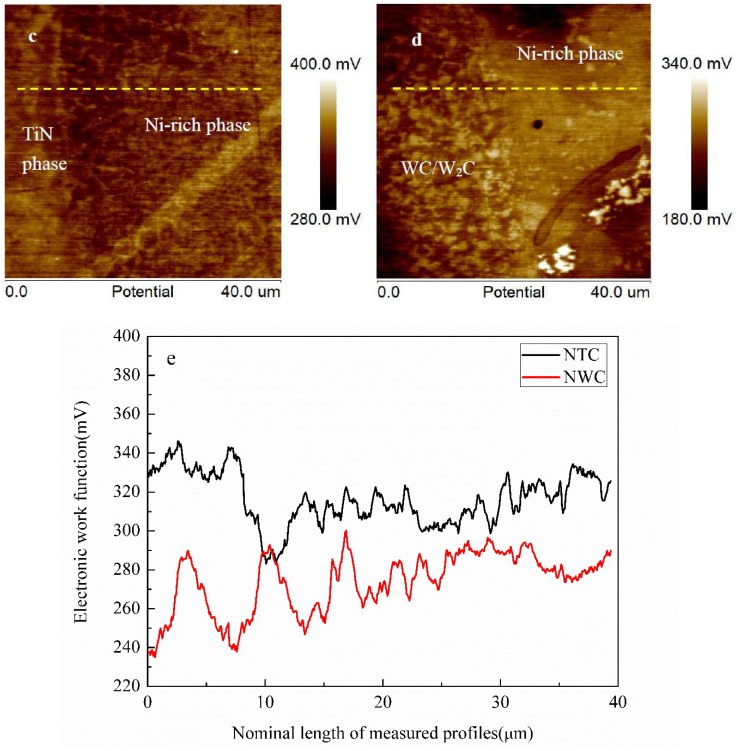
AFM images of the cross-sections of: (**a**,**c**) NTC coating, and (**b**,**d**) NWC coating. (**e**) EWF line profiles of coatings corresponding to the dashed lines in Figure 5**c**,**d**.

The EWF test was also used to survey the NTC and NWC coatings. A higher work function usually corresponds to a stronger atomic bond, stronger interfacial bond, higher hardness, and resistance to wear [27,28]. The EWF and morphology maps are shown in Figure 5. The height difference of the TiN/TiN_0.3_ and Ni-rich phase was 340 nm in the NTC coating, and of the WC/W_2_C and Ni-rich phase was 240 nm in the NWC coating (Figure 5a,b). The height difference was due to the difference in grind resistance between the hard particles and the matrix phase. The matrix was the Ni-rich phase, and height differences can fully illustrate that the grind resistance of TiN in the NTC coating and WC in the NWC coating was different. According to experimental measurements, the TiN phase in this kind of composite coating has higher grind resistance than the WC/W_2_C phase. This is in excellent agreement with the wear test results. In addition, the NTC coating domain possesses higher EWF than the NWC coating (Figure 5e). This suggests that the NTC coating possesses higher hardness, stronger resistance to wear, and stronger atomic bond than the NWC coating [27,28]. This is likely due to TiN/TiN_0.3_ being formed by in situ reaction with high hardness and good combination with the matrix.

**Figure 6:**
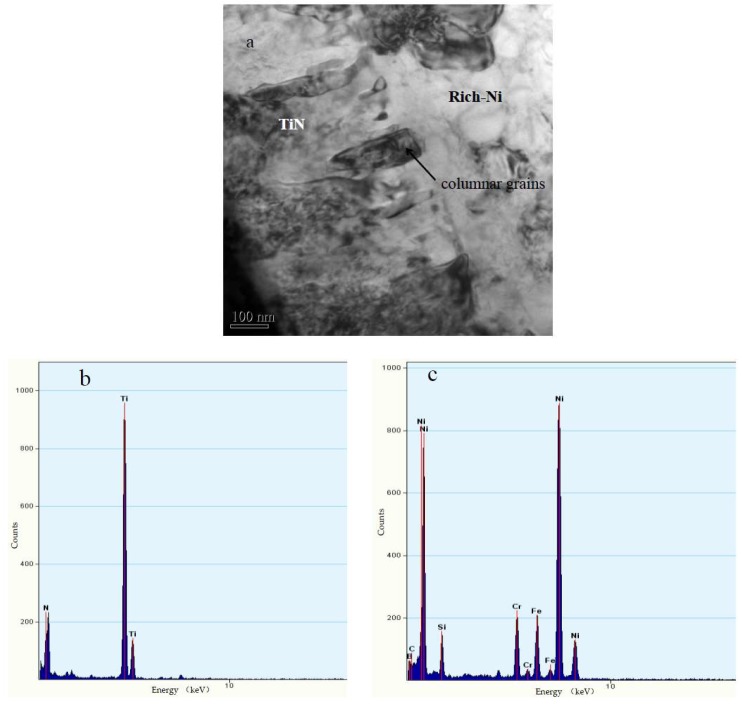
(**a**) TEM image of interfaces between TiN and γ-Ni layer; (**b**,**c**) EDS spectra.

Figure 6 shows the TEM (Hitach Ltd., Tokyo, Japan) morphologies of the NTC coating. Figure 6a shows the interface between the Ti-rich phase and Ni-rich phase, and Figure 6b,c, respectively, show the EDS spectrum of the Ti-rich and Ni-rich areas in Figure 6a. The Ti-rich area and Ni-rich area were the TiN phase and Ni solid solution phase, respectively. The TiN phase and nickel-based solid solutions were tightly bonded together, and the Ti-rich phase was composed of elongated columnar grains with a diameter of about 100 nm. The completely melted Ti powder reacted in situ with nitrogen, and the resulting TiN/TiN_0.3_ nucleated and grew in the rich-Ni matrix during the spraying process. Therefore, the TiN/TiN_0.3_ was composed of nanosized crystal grains, which was explained in detail in our previous work [24,29]. However, WC particles exhibited a semimelted or unmelted state in the composite coating due to their high melting point, so that the WC retained its original micron-scale morphology.

### 3.2. Microhardness

**Figure 7:**
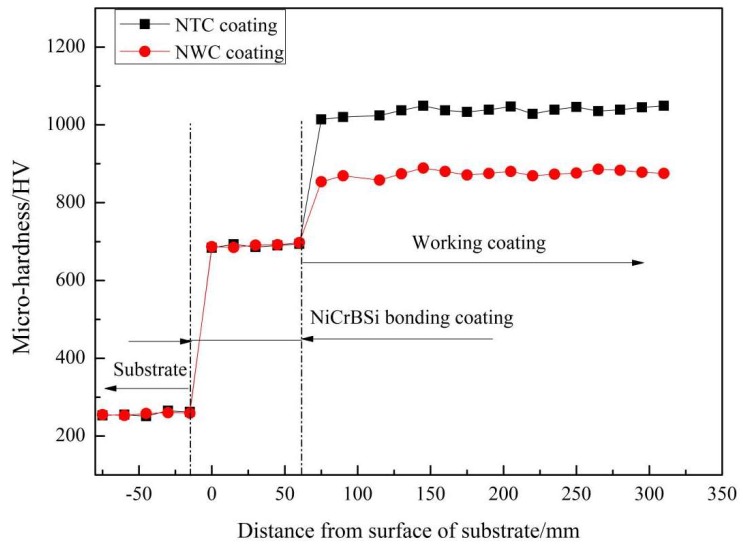
Microhardness of NTC and NWC coatings.

Figure 7 shows the microhardness of the NTC and NWC coating cross-sections. The NTC coating had microhardness exceeding 1000 HV, while the NWC coating had microhardness of around 850 HV. Because the hardness of WC is higher than that of TiN, the hardness of the NWC coating should, theoretically, be higher than that of the NTC coating. As a matter of fact, the microhardness of the NTC coating was approximately 100 HV higher than that of the NWC coating. This is because WC decomposed to lower-hardness W_2_C during the spraying process, and the WC phase did not bond tightly with the matrix. The in situ reaction of TiN tightly binding with the matrix resulted in an enhanced strengthening effect in the coating. Another reason the hardness of TiN in composite coating was higher was that the TiN phase had a nanograin structure.

**Figure 8:**
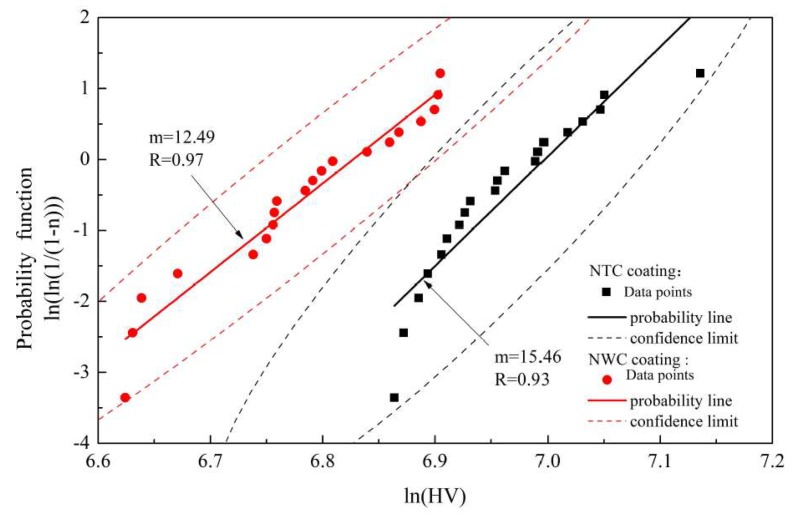
Weibull distribution plot of the microhardness of NTC and NWC coatings.

Simply evaluating the hardness of the composite coating by average hardness does not yield reliable results. The Weibull distribution is widely used with reliability analysis and discrete data processing. In this paper, the hardness of the composite coating was analyzed with a two-parameter Weibull distribution model. Figure 8 shows the confidence limit and the Weibull distribution plots of the Vickers microhardness of two composite coatings, where R represents the correlation coefficient between the measuring point and the regression line of the NTC and NWC coatings, and m is the slope of Weibull curve and represents the dispersion degree of microhardness [30]. The confidence limits of the NTC and NWC coatings are shown by black and red dashed lines, respectively. In general, the larger the slope of the Weibull curve, the higher the correlation coefficient and the better the correlation of the microhardness data.

Under a confidence level of 0.99, the values of R for the NTC and NWC coatings were 0.93 and 0.97, respectively. The values of b were 15.46 and 12.49, respectively. The microhardness of the NTC coating had a lower degree of dispersion than the NWC coating. Since all data points are in the confidence limits, the evaluation of the microhardness is reliable. Therefore, the microhardness of the NTC coating was superior to that of the NWC coating under an indentation load of 200 g.

### 3.3. Wear Resistance 

**Figure 9:**
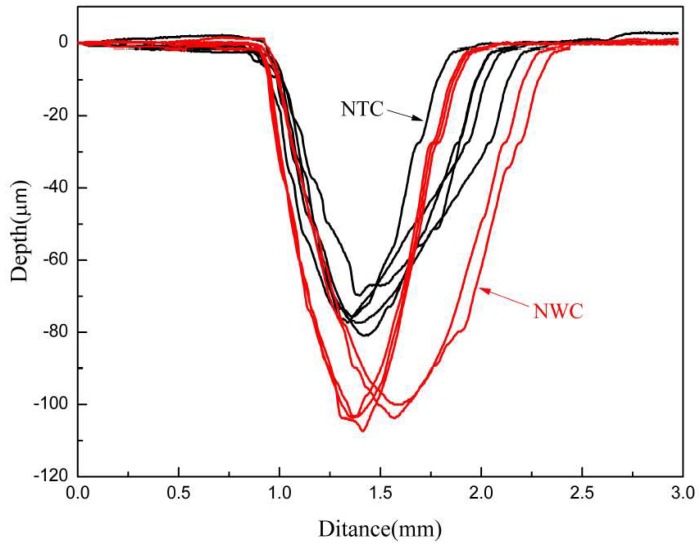
Wear profiles of NTC and NWC coatings.

Figure 9 presents the wear profiles of the composite coating, and Table 3 lists the corresponding results. The NWC coating had a larger profile and the corresponding wear scar width and depth were larger than those of the NTC coating (Table 3). Wear volume of the NTC coating was reduced by 45% compared to the NWC coating. This suggests that the NTC coating had excellent wear resistance.

**Figure 10:**
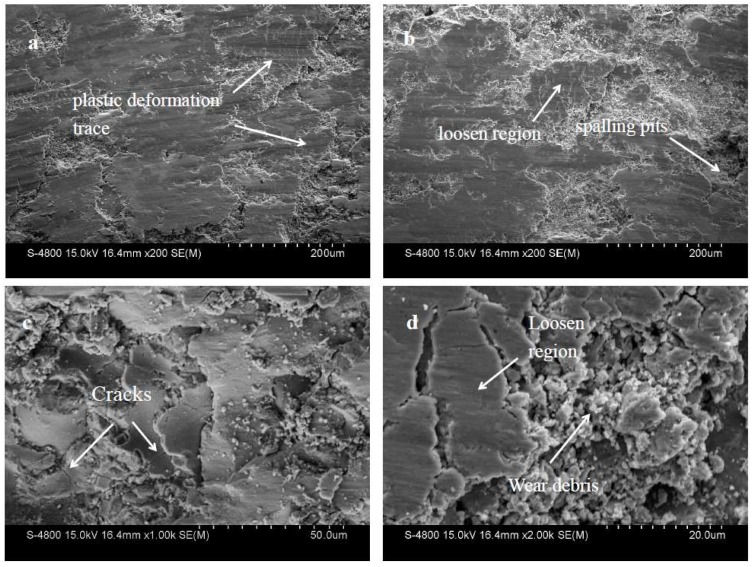
Wear morphology of NTC coating.

Figure 10 presents the worn morphology of the NTC coating. Some fracture chipping pits and plastic deformation traces were observed on the worn surface of the NTC coating, as shown in Figure 10a. This indicates that the main surface damage patterns of the NTC coating were microplastic deformation, material subsidence in the loosened region, microcracks, and spalling pits. The reason for these phenomena is that the softer γ-Ni phase supports the harder TiN. When the surface is locally subjected to high loading, the softer γ-Ni phase begins to plastically deform, while the harder TiN is difficult to plastically deform. Cracks start to sprout and expand. With the continuing effect of loading, the broken pieces peel off the composite coating and leave a peeling pit. There were cracks in some areas of the sample surface where γ-Ni broke from these positions and peeled off the surface. The wear mechanism of the NTC coating was mainly plastic deformation and lamellar peeling. 

**Figure 11:**
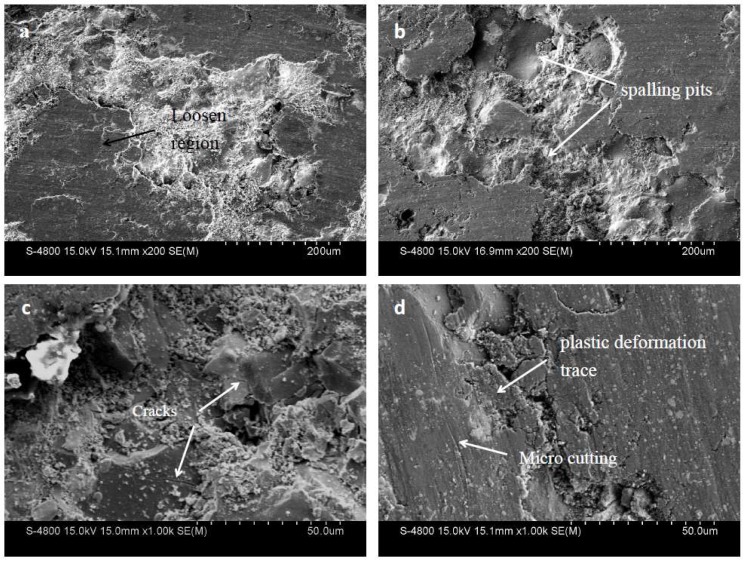
Wear morphology of NWC coating.

Figure 11 shows the worn surfaces of the NWC coating. Plastic deformation traces, loosened region, and microcutting were very obvious. The high microhardness of the WC material resulted in an increased ability of the coating to resist microcutting [31]. Therefore, microcutting trace was relatively shallow. Spalling pits can clearly be observed. Compared with the NTC coating, spalling pits of the NWC coating were bigger and deeper. If the shear force was less than the bonding force between the hard phase and the matrix metal during wear, the hard particles could protect the matrix phase from the friction of the friction pair [32]. If the shear force was greater than the bonding force between the hard phase and the matrix metal, the hard particles would peel off the matrix and participate in the wear, resulting in a greater amount of wear on the coating [33]. 

According to the above description, the WC/W_2_C phase is more likely to peel off the matrix metal than the Ti-rich phase, because the bonding strength of the TiN and Ni alloy phases is stronger than that of the WC/W_2_C and alloy phases. The detached WC hard particles participated in the wear process as an abrasive, which in turn exacerbated wear [34]. This also explains why the wear volume of the NTC coating will be less than that of the NWC coating.

## 4. Conclusions

(1) The nano-TiN phase in NiCrBSi–TiN composite (NTC) coating was obtained through in situ reaction between Ti powder and nitrogen in the process of plasma spraying. The NTC coating had typical lamellar structure due to sprayed particles fully melting and flattening during plasma spraying, and displayed good integrated condition, in close contact with the Ni-based alloy matrix. Therefore, the NTC coating had a denser structure than the NiCrBSi–WC composite (NWC) coating.

(2) The average microhardness of the NTC coating exceeded 1000 HV, while the microhardness of the NWC coating was around 850 HV. The reason is that the TiN phase in the NTC coating had nanostructure and close interface with the Ni-based alloy matrix. WC decomposed to lower hardness W_2_C during the spraying process, and the WC phase had micron structure.

(3) The sliding wear resistance of the NTC coating was significantly better than that of the NWC coating. The average wear loss volume was 0.8118 mm^3^ and 1.4772 mm^3^, respectively. The wear mechanism of both composite coatings included plastic deformation and lamellar peeling. The good bonding between the TiN hard phase and the NiCrBSi alloy matrix helped to increase the mechanical properties of the composite coating.

## Figures and Tables

**Table 1:** Chemical composition of NiCrBSi powder (wt.%).

Element	Ni	Cr	B	Si	Fe	C
wt.%	Bal.	19.0–21.0	3.3–4.2	3.3–4.2	14.0–16.0	0.5–0.7

**Table 2:** EDS analysis results of different marked areas in Figure 3 (wt.%).

Coating	Point	Ni	Cr	Si	Fe	Ti	N	W	C
NTC	A	69.45 ± 0.73	17.7 ± 1.35	0.83 ± 0.05	11.2 ± 1.39				
NTC	B					63.75 ± 2.79	37.35 ± 1.98		
NWC	C	66.07 ± 1.75	18.2 ± 1.56	3.47 ± 0.09	12.22 ± 1.4				
NWC	D							61.62 ± 3.87	38.38 ± 3.87

**Table 3:** Sliding wear results for the coatings.

Samples	Wear Width (mm)	Wear Depth (μm)	Wear Volume (mm^3^)
NTC	1.0985	66.88	0.8118
NWC	1.2871	99.115	1.4772
